# Predictive machine learning model for microvascular invasion identification in hepatocellular carcinoma based on the LI-RADS system

**DOI:** 10.3389/fonc.2022.1021570

**Published:** 2022-11-08

**Authors:** Xue Yang, Guoqing Shao, Jiaojiao Liu, Bin Liu, Chao Cai, Daobing Zeng, Hongjun Li

**Affiliations:** ^1^ Department of Radiology, Beijing You’an Hospital, Capital Medical University, Beijing, China; ^2^ Department of Radiology, Xuzhou Central Hospital, Xuzhou, China; ^3^ Department of Radiology, Civil Aviation General Hospital, Beijing, China; ^4^ General Surgery Department, Beijing Youan Hospital, Capital Medical University, Beijing, China; ^5^ Clinical Center for Liver Cancer, Capital Medical University, Beijing, China

**Keywords:** hepatocellular carcinoma, microvascular invasion, liver imaging reporting and data system, machine learning, contrast enhanced computed tomography

## Abstract

**Purposes:**

This study aimed to establish a predictive model of microvascular invasion (MVI) in hepatocellular carcinoma (HCC) by contrast-enhanced computed tomography (CT), which relied on a combination of machine learning approach and imaging features covering Liver Imaging and Reporting and Data System (LI-RADS) features.

**Methods:**

The retrospective study included 279 patients with surgery who underwent preoperative enhanced CT. They were randomly allocated to training set, validation set, and test set (167 patients vs. 56 patients vs. 56 patients, respectively). Significant imaging findings for predicting MVI were identified through the Least Absolute Shrinkage and Selection Operator (LASSO) logistic regression method. Predictive models were performed by machine learning algorithm, support vector machine (SVM), in the training set and validation set, and evaluated in the test set. Further, a combined model adding clinical findings to the radiologic model was developed. Based on the LI-RADS category, subgroup analyses were conducted.

**Results:**

We included 116 patients with MVI which were diagnosed through pathological confirmation. Six imaging features were selected about MVI prediction: four LI-RADS features (corona enhancement, enhancing capsule, non-rim aterial phase hyperehancement, tumor size) and two non-LI-RADS features (internal arteries, non-smooth tumor margin). The radiological feature with the best accuracy was corona enhancement followed by internal arteries and tumor size. The accuracies of the radiological model and combined model were 0.725–0.714 and 0.802–0.732 in the training set, validation set, and test set, respectively. In the LR-4/5 subgroup, a sensitivity of 100% and an NPV of 100% were obtained by the high-sensitivity threshold. A specificity of 100% and a PPV of 100% were acquired through the high specificity threshold in the LR-M subgroup.

**Conclusion:**

A combination of LI-RADS features and non-LI-RADS features and serum alpha-fetoprotein value could be applied as a preoperative biomarker for predicting MVI by the machine learning approach. Furthermore, its good performance in the subgroup by LI-RADS category may help optimize the management of HCC patients.

## 1 Introduction

Hepatocellular carcinoma (HCC) represents the third leading cause of cancer-related mortality worldwide and remains a growing tendency (1). Microvascular invasion (MVI) indicates the frequent recurrence, extrahepatic metastases, and poor prognosis after surgical resection and transplantation in HCC patients (2–4). It is critical to detect the presence of MVI at the time of the diagnosis of liver lesion, which impact the choice involving wide margin hepatectomy or combined with intraoperative electron radiotherapy, or liver transplantation, or tumor ablation (3–6). Thus, MVI urgently requires preoperative prediction to apply the optimal therapeutic strategy, rather than being diagnosed through microscopic examination of a pathological specimen after hepatectomy (7, 8). Adequate number of sampling sites (NUSS) and sampling location affect the detection rate of MVI. Chen et al. suggested that at least four to eight NUSS within paracancerous parenchyma ≤1 cm were needed for assessing MVI according to tumor size (9). Thus, pathological biopsy is likely to increase false-negative rates before surgery due to sampling errors and number of NUSS.

With increasing attention to MVI, serum markers (i.e., AFP) and imaging features as non-invasive examinations before surgery, such as internal arteries and tumor margin, have been used to predict MVI (10–12). However, prior studies showed heterogeneous standards and varied accuracies, which require further research (10–12). The Liver Imaging Reporting and Data System (LI-RADS) has been recognized for HCC diagnosis (13). Regarding its advantages of standardized criteria and being easy to generalize, few studies have also attempted to explore the relationship between LI-RADS features and MVI in HCC. For example, ancillary features of LI-RADS including absence of nodule-in-nodule architecture, coronal enhancement, mosaic architecture, and LR-M features including marked diffusion restriction and rim arterial enhancement have been reported as predictive factors of MVI (12, 14–16). These studies are mainly derived from magnetic resonance imaging (MRI) features, whereas there was shortage of computed tomography (CT)-driven results (12, 14–19). Additional studies are needed to determine whether predictive MRI features are still applicable for CT. Compared with MRI, CT has some advantages which are shorter scanning time and lower cost, more stability, and not being limited by the presence of metal objects implanted in the human body. Therefore, research of CT deserves further study. This may also help in expanding the power of LI-RADS features in clinical decision making and obtaining predictive models of MVI based on standardized CT imaging features.

Machine learning has been used to extract radiomics features and develop corresponding models for MVI prediction (20–22). Although models with radiomics features presented good performance, the interpretability and generalizability limit clinical application. The machine learning approach could identify complicated interactions among predictive factors and be able to avoid overfitting as well as improve the performance of the model (23, 24). However, it is underused in interpretable imaging features. The combination of interpretable imaging features and machine learning method may find functional information and aid in the identification of MVI.

By machine learning algorithms, our purpose is to establish and evaluate visibly standardized CT feature-based models for MVI detection. Additionally, subgroup analyses are used to evaluate model performance for risk stratification of HCC patients according to LI-RADS category.

## 2 Materials and methods

The single-center study was approved by the ethical committees of the Beijing Youan Hospital, Capital Medical University, and complied with the ethical guidelines of the Declaration of Helsinki. Owing to the retrospective nature of our study, the institutional review board waived the patients’ informed consent.

### 2.1 Patient selection

**Figure 1:**
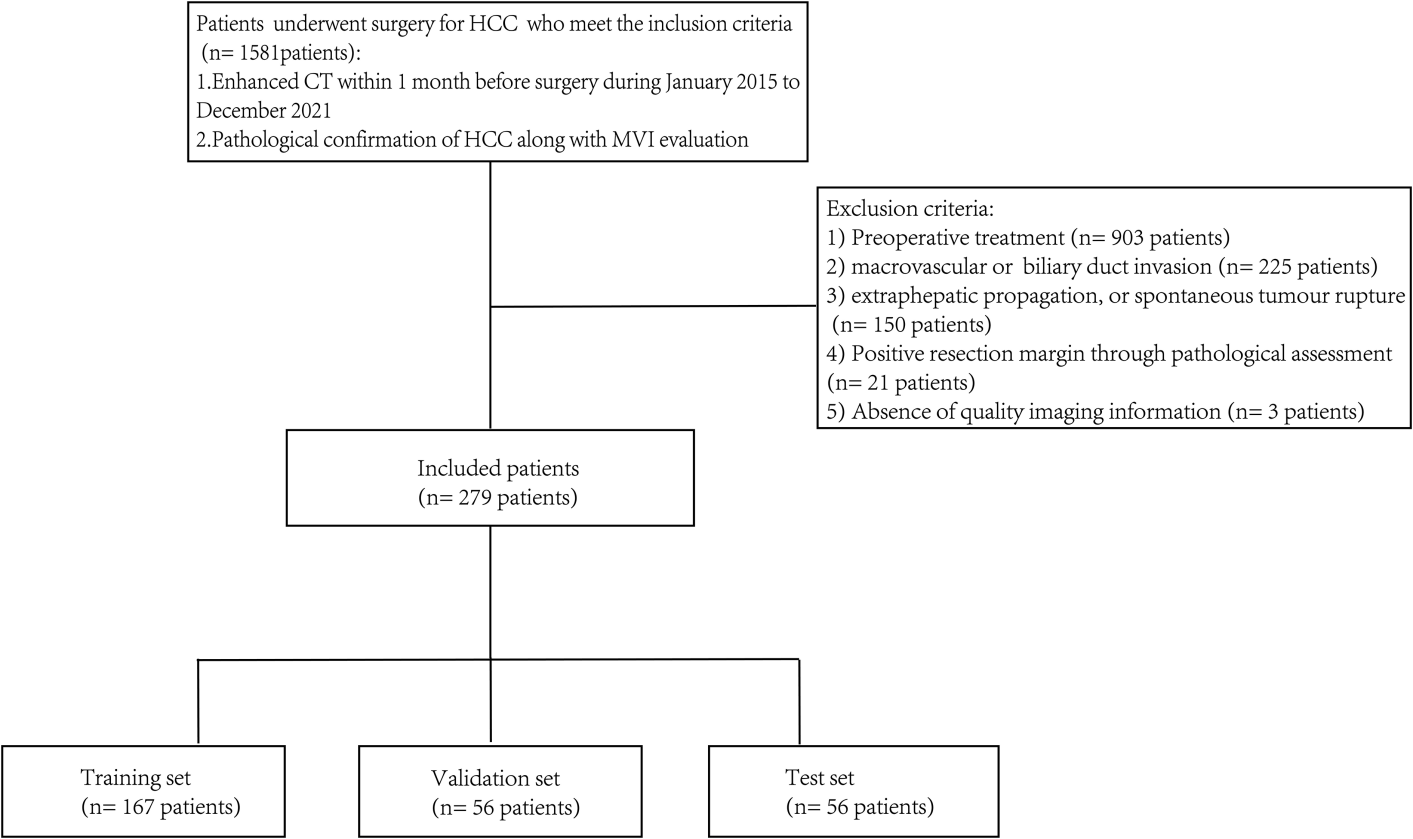
The flowchart for patient selection.

Consecutive adult patients were recruited from January 2015 to December 2021. A total of 1,581 patients were screened according to the selection criteria, which were as follows: 1) diagnosed by histological evaluation in accordance with the American Association for the Study of Liver Diseases (AASLD) or European Association for the Study of the Liver (EASL) guidelines for HCC (25); 2) underwent enhanced CT examinations within 1 month before surgery; 3) patients whose state of MVI was evaluated by histological results. The excluded criteria included the following: 1) any preoperative treatment; 2) macrovascular or biliary duct invasion; 3) positive resection margin through pathological assessment; 4) extrahepatic propagation or spontaneous tumor rupture; 5) absence of quality imaging information. A flowchart for subjects is provided in **Figure 1**. Finally, we selected 279 patients, who were randomly allocated to training set, validation set, and test set through a 6:2:2 split (training set: 167 patients, validation set and test set: both 56 patients).

### 2.2 Imaging protocol

A contrast-enhanced multiphase CT examination was performed on a 64-multidetector CT scanner (GE LightSpeed VCT, USA). Intravenous administration of an iodine contrast agent (370 mg I/ml of iopromide 370, Schering, Berlin, German) with a dose of 1.5 ml/kg was provided at a rate of 3 ml/s using the automatic bolus-tracking program. The experienced radiology technician controlled the celiac artery attenuation and placed the region of interest in the abdominal aorta. The trigger threshold level-based SmartPrep contrast was set at 100 Hounsfield units. The contrast-enhanced CT scanning protocol is summarized in the **Supplemental Table 1**.

### 2.3 Features

#### 2.3.1 Imaging features

Three board-certified gastrointestinal radiologists independently and retrospectively reviewed the enhanced CT images, who were informed the diagnosis of HCC, but unaware of other outcomes of pathologic examinations and clinical information. For multifocal HCC, the lesion with the largest diameter was selected for assessment. According to LI-RADS version 2018 (13), reviewers determined the imaging characteristics of tumors: major features (non-rim arterial phase hyperenhancement (APHE), non-peripheral washout, tumor size, and enhancing capsule), ancillary features (corona enhancement, nodule-in-nodule architecture, mosaic architecture, blood products in mass), and LR-M features (targetoid features including rim APHE and peripheral washout and delayed enhancement, intratumor necrosis). Moreover, non-LI-RADS features based on previous studies were also evaluated: tumor number, internal arteries, non-smooth tumor margin, peritumoral hypoattenuating halo, and tumor–liver difference. **Supplemental Table 2** shows the definition of imaging features in detail. The LI-RADS category was also allocated to tumor lesions. When there was a difference of opinion about the evaluation of any imaging characteristic, a second review was done until agreement was reached.

#### 2.3.2 Clinical features

The clinical characteristics included age, gender, etiology of chronic liver disease, Child–Pugh grade, and blood variables (shown in **Table 1**).

**Table 1:** Participant characteristics.

Variable	Training set (n = 167)			Validation set (n = 56)			Test set (n = 56)		
	MVI positive	MVI negative	*P* value	MVI positive	MVI negative	*P* value	MVI positive	MVI negative	*P* value
Demographic variables									
Age (years)	54.99 ± 10.77	57.30 ± 9.07	0.135	58 (49-64)	58 (48-64)	0.739	54.09 ± 10.56	55.91 ± 12.00	0.560
Gender (male (%))	52 (74.3%)	84 (86.6%)	0.043*	20 (87%)	27 (81.8%)	0.723	15 (65.2%)	30 (90.9%)	0.037*
Blood signatures									
Etiology (HBV (%))	64 (91.3%)	84 (86.6%)	0.332	21 (91.3%)	28 (84.8%)	0.688	22 (95.7%)	31 (93.9%)	1.000
Child–Pugh (A (%))	68 (97.1%)	93 (95.8%)	1.000	22 (95.7%)	33 (100%)	0.411	23 (100%)	31 (93.9%)	0.507
AFP (>400 ng/ml (%))	21 (30%)	10 (10.3%)	0.001*	7 (30.4%)	4 (12.1%)	0.170	7 (30.4%)	3 (9.1%)	0.073
ALT (>50 U/l (%))	14 (20%)	14 (14.4%)	0.342	5 (21.7%)	5 (15.2%)	0.725	3 (13%)	6 (18.2%)	0.723
AST (>40 U/l (%))	22 (31.4%)	21 (21.6%)	0.154	7 (30.4%)	9 (27.3%)	1.000	5 (21.7%)	6 (18.2%)	0.746
TBIL (>21 µmol/l (%))	13 (18.6%)	25 (25.8%)	0.273	7 (30.4%)	11 (33.3%)	1.000	7 (30.4%)	9 (27.3%)	0.797
ALB (<40 g/l (%))	28 (40%)	34 (35.1%)	0.514	12 (52.2%)	13 (39.4%)	0.344	8 (34.8%)	12 (36.4%)	1.000
PT (>12 s (%))	13 (18.6%)	25 (25.8%)	0.273	7 (30.4%)	9 (27.3%)	0.797	3 (13%)	8 (24.2%)	0.496
PLT (<125 * 10^9^/l (%))	18 (25.7%)	38 (39.2%)	0.069	9 (39.1%)	14 (42.4%)	0.805	4 (17.4%)	11 (33.3%)	0.185
WBCs (<3.5 * 10^12^/l (%))	6 (8.6%)	15 (15.5%)	0.185	2 (8.7%)	5 (15.2%)	0.688	3 (13.0%)	4 (12.1%)	1.000
DNA load (>10^4^ IU/ml (%))	12 (17.1%)	21 (21.6%)	0.471	4 (17.4%)	8 (24.2%)	0.743	4 (17.4%)	7 (21.2%)	1.000
Radiologic features									
Tumor number (single (%))	64 (91.4%)	95 (97.9%)	0.070	22 (95.7%)	30 (90.9%)	0.636	23 (100%)	32 (97%)	1.000
Non-smooth tumor margin (%)	55 (78.6%)	47 (48.5%)	0.000*	21 (91.3%)	21 (63.6%)	0.019*	19 (82.6%)	18 (54.5%)	0.029*
Internal arteries (%)	53 (75.7%)	42 (43.3%)	0.000*	17 (73.9%)	11 (33.3%)	0.003*	18 (78.3%)	15 (45.5%)	0.014*
Peritumoral hypoattenuating halo (%)	10 (14.3%)	17 (17.5%)	0.575	3 (13%)	3 (9.1%)	0.681	7 (30.4%)	2 (6.1%)	0.024*
Tumor-liver difference (%)	12 (17.1%)	14 (14.4%)	0.634	8 (34.8%)	4 (12.1%)	0.054	7 (30.4%)	5 (15.2%)	0.200
LI-RADS features									
Major features									
Non-rim AP hyperenhancement (%)	50 (71.4%)	81 (83.5%)	0.061	15 (65.2%)	27 (81.8%)	0.158	14 (60.9%)	25 (75.8%)	0.233
Non-peripheral “washout” (%)	52 (74.3%)	82 (84.5%)	0.101	15 (65.2%)	28 (84.8%)	0.087	14 (60.9%)	24 (72.7%)	0.350
Enhancing capsule (%)	61 (87.1%)	74 (76.3%)	0.079	20 (87%)	28 (84.8%)	1.000	22 (95.7%)	30 (90.9%)	0.636
Tumor size (>5 cm (%))	28 (40%)	12 (12.4%)	0.000*	11 (47.8%)	4 (12.1%)	0.003*	10 (43.5%)	7 (21.2%)	0.075
Ancillary features									
Corona enhancement (%)	32 (45.7%)	18 (18.6%)	0.000*	12 (52.2%)	9 (27.3%)	0.058	13 (56.5%)	6 (18.2%)	0.003*
Nodule-in-nodule architecture (%)	0	0	1.000	0	0	1.000	0	1 (3.0%)	1.000
Mosaic architecture (%)	13 (18.6%)	12 (12.4%)	0.268	9 (39.1%)	4 (12.1%)	0.019*	2 (8.7%)	5 (15.2%)	0.688
Blood products in mass (%)	1 (1.4%)	0	0.419	0	1 (3%)	1.000	0	0	1.000
LR-M features									
Targetoid feature (%)	17 (24.3%)	13 (13.4%)	0.071	8 (34.8%)	5 (15.2%)	0.087	9 (39.1%)	7 (21.2%)	0.144
AP rim hyperenhancement (%)	16 (22.9%)	12 (12.4%)	0.073	8 (34.8%)	5 (15.2%)	0.087	9 (39.1%)	7 (21.2%)	0.144
PVP/DP peripheral “washout” (%)	16 (22.9%)	11 (11.3%)	0.046*	8 (34.8%)	5 (15.2%)	0.087	9 (39.1%)	6 (18.2%)	0.082
Delayed central enhancement (%)	2 (2.9%)	8 (8.2%)	0.195	2 (8.7%)	1 (3%)	0.562	0	3 (9.1%)	0.261
Intratumor necrosis (%)	36 (51.4%)	36 (37.1%)	0.065	17 (73.9%)	12 (36.4%)	0.006*	16 (69.6%)	15 (45.5%)	0.074

HBV, hepatitis B virus; AFP, alpha-fetoprotein; ALT, alanine aminotransferase; AST, aspartate aminotransferase; TBIL, total bilirubin; ALB, albumin; PT, prothrombin time; PLT, platelet count; WBC, white blood cell; LI-RADS, Liver Imaging Reporting and Data System; AP, arterial phase; PVP, portal vein phase; DP, delayed phase.

*p < 0.05.

### 2.4 Statistical analysis

Feature selection could affect learning accuracy and result in comprehensibility. We applied the Least Absolute Shrinkage and Selection Operator (LASSO) for imaging feature selection. Support vector machine (SVM) was used to develop the machine learning model for MVI prediction in HCC. In addition, continuous blood signatures were translated into categorical variables and compared *via* chi-squared test or Fisher’s exact test (with *p* < 0.05). Demographic variables were compared by chi-squared test or Student t test (*p* < 0.05). Then, we found the significant clinical variables based on logistic regression (*p* < 0.05) which were combined with radiologic features to develop combined model.

#### 2.4.1 Feature preprocessing and selection

Minimization of the empirical error penalized by a regularization term, namely, totality of the empirical error (loss term) and regularization term (penalty term), is applied to construct a sparse learning model for feature selection (24). The LASSO logistic regression algorithm is frequently used as the standard sparse regression approach on the basis of the regularization framework. The regularization parameter λ is the balance between the loss term and the penalty term (26).

We standardized features with the z score. The value of regularization parameter λ was selected *via* 10-fold cross-validation in the training set, which resulted in the sparsest model remaining within one standardized error of the minimum loss. Imaging features were selected which yielded non-zero coefficients (27). The multicollinearity of the significant attributes were evaluated using the variation inflation factor (VIF<5).

#### 2.4.2 Training and evaluation processes of the predictive model for MVI

We developed radiological predictive model using SVM and optimized hyperparameters through learning curves and grid searches in the training set and validation set. Furthermore, a combined model using features in the radiologic model with a serum alpha-fetoprotein (AFP) value was also used in the training set and the validation set and assessed in the test set.

The sensitivity, specificity, accuracy, positive predictive value (PPV), and negative predictive value (NPV) of the MVI were compared using the McNemar test. Areas under the curve (AUC) of two models for MVI detection were compared by the Delong test. Moreover, we made the decision curve analysis (DCA) counting the net benefits under different threshold probabilities, which indicated the clinical relevance of the prediction model (28). The calibration curve was used to evaluate the goodness of models.

We defined three thresholds from the results of the training set: Youden’s index, high specificity threshold (95% specificity), and high sensitivity threshold (95% sensitivity). Performance matrices were evaluated according to three thresholds of MVI probabilities in the test set, namely, the whole set, LR 4/5 group, and LR-M group. Statistical analyses were performed using Python ver. 3.8 and SPSS software ver. 26.0. Inter-reader agreement among three observers for the CT features was assessed by a Fleiss’ kappa value of ≤0.20 which means poor level, 0.21–0.40 which is fair level, 0.41–0.60 which is moderate level, 0.61–0.80 which is substantial level, and ≥0.80 which means nearly best level.

## 3 Results

### 3.1 Patients’ characteristics

A total of 279 patients were enrolled in this study (**Table 1**). One hundred sixty-seven patients, 56 patients, and 56 patients constituted the training set, validation set, and test set, which involved 70 (41.9%), 23 (41.1%), and 23 (41.1%) patients with presence of MVI, respectively (*p* = 0.990). The clinical variables were similar among the training set, validation set, and test set (*p* = 0.278–0.97). Between MVI-positive patients and MVI-negative patients, clinical factors including AFP and gender were significantly different in the training set and the test set, separately. Many radiologic features were also different between the MVI-positive group and MVI-negative group in the training, validation, and test sets which are shown in detail in **Table 1**.

### 3.2 Feature selection

**Figure 2:**
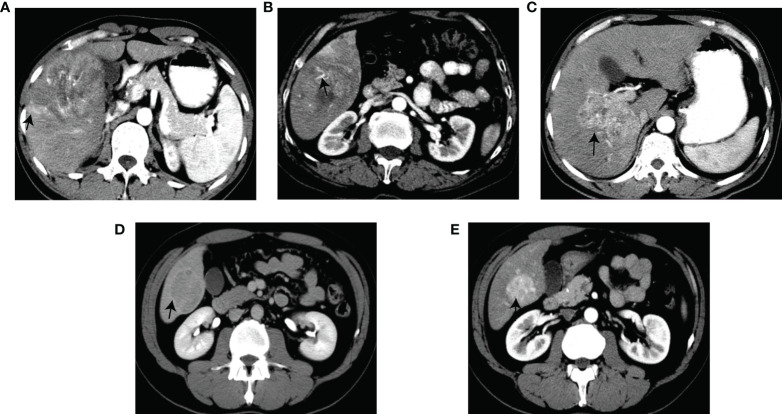
Graphical illustration of the selected features. Corona enhancement **(A)**. Internal arteries **(B)**. Non-smooth margin **(C)**. Enhancing capsule **(D)**. Non-rim AP hyperenhancement **(E)**.

**Figure 3:**
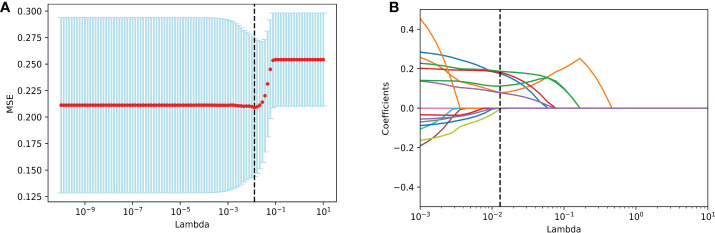
Imaging feature selection for MVI identification in the training set by LASSO logistic regression. **(A)** Selection of tuning λ in the LASSO model through 10-fold cross-validation. Vertical black lines define the best values of λ that provide the best fit. **(B)** LASSO coefficient profiles of all imaging features for MVI presence. Vertical black lines are plotted at the value selected in **(A)**. Imaging features with non-zero coefficients are indicated. MVI, microvascular invasion; LASSO, Least Absolute Shrinkage and Selection Operator.

Six radiological features were selected *via* the LASSO logistic regression approach with the optimal λ (λ = 0.0129). There are four LI-RADS features (corona enhancement, enhancing capsule, non-rim aterial phase hyperehancement, tumor size) and two non-LI-RADS features (internal arteries, non-smooth tumor margin) (**Figure 2**). Misclassification errors and coefficients are shown in **Figure 3**. The VIF values of those features ranged 1.10–1.49 (all <5), which were proved without multicollinearity. Their Fleiss’ kappa values ranged from 0.331-0.974 (listed in **Supplementary Table 3**). At the aspect of blood signatures and demographic variables, we only found that AFP was related to MVI using multivariate logistic regression in the training set (*p* < 0.05) (**Supplementary Table 4**).

### 3.3 Development and validation of MVI prediction models

**Figure 4:**
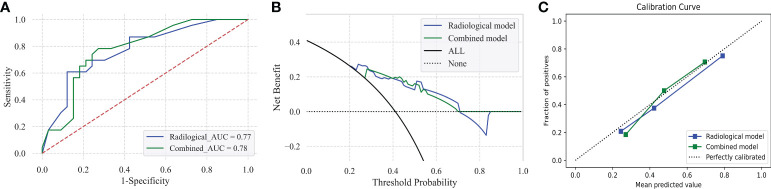
Predictive performance of the radiological model and combined model for MVI prediction in HCC patients in the test set **(A–C)**. Receiver operating characteristic (ROC) curves of radiological model and combined model in the test set **(A)**. Decision curve graphics (DCA) of the radiological model and combined model in the test set **(B)**. Calibration curve graphics of predicted risk based on the radiological model and combined model in the test set **(C)**.

A radiological model using the SVM approach that integrated corresponding radiologic predictors was built and optimized in the training set and validation set, which presented an AUC and accuracy of 0.795/0.793 and 0.725/0.714, respectively (**Supplementary Table 5**). The AUC and accuracy of the radiological model in the test model were 0.775 and 0.714 (**Table 2**). The comparison predictive performance between radiological model and combined model which was added with AFP presented no significant difference among the three sets (AUC: *p* = 0.08–0.569, accuracy: *p* = 0.125–1.000, sensitivity: *p* = 0.065–1.000, specificity: *p* = 0.625–1.000). However, the combined model obtained a better benefit than the radiological model for MVI probability examination by DCA (**Figure 4** and **Supplementary Figure 1**). Notably, a calibration curve of the combined model demonstrated a better calibration than that of the radiologic model, which indicated a general underestimation of MVI risk in HCC (**Figure 4**). We also conducted subgroup analyses using both the radiological model and combined model in the test set. The combined model maintained good performance in both the LR-4/5 and LR-M groups rather than radiological model (**Table 2**).

**Table 2:** Predictive performance of models for MVI identification in the test set.

	AUC	Sensitivity	Specificity	PPV	NPV	Accuracy
Whole test set						
Radiological model	0.775	0.61	0.79	0.67	0.74	0.714
Combined model	0.778	0.65	0.79	0.68	0.76	0.732
LR-4/5 group						
Radiological model	0.782	0.57	0.85	0.67	0.79	0.750
Combined model	0.760	0.50	0.85	0.64	0.76	0.725
LR-M group						
Radiological model	0.643	0.67	0.57	0.67	0.57	0.625
Combined model	0.762	0.89	0.57	0.73	0.80	0.750

MVI, microvascular invasion; AUC, area under the curve; PPV, positive predictive value, NPV, negative predictive value.

**Figure 5:**
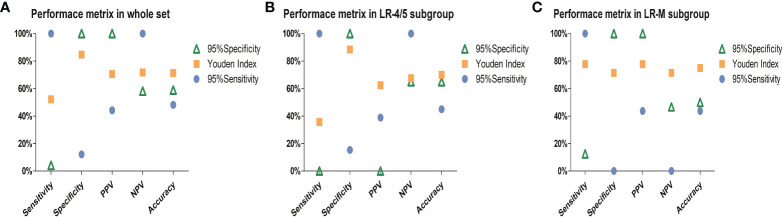
Charts exhibit performance metrics of the combined model in the **(A)** whole test set, **(B)** LR-4/5 subgroup, and **(C)** LR-M subgroup. PPV, positive predictive value; NPV, negative predictive value. Ninety-five percent sensitivity threshold and high specificity threshold of combined model were 0.27026 and 0.6954, respectively.

Furthermore, the Youden index, high-sensitivity threshold, and high-specificity threshold of the combined model were calculated as 0.59, 0.27026, and 0.6954 in the training set, respectively. Based on the Youden index, the sensitivity, specificity, PPV, NPV, and accuracy of the test set were 52.2%, 84.8%, 70.6%, 71.8%, and 71.4%, respectively (**Figure 5**). In the LR-4/5 subgroup, a sensitivity of 100% and an NPV of 100% were obtained through the high-sensitivity threshold. In the LR-M subgroup, a specificity of 100% and a PPV of 100% were acquired from the high-specificity threshold.

**Figure 6:**
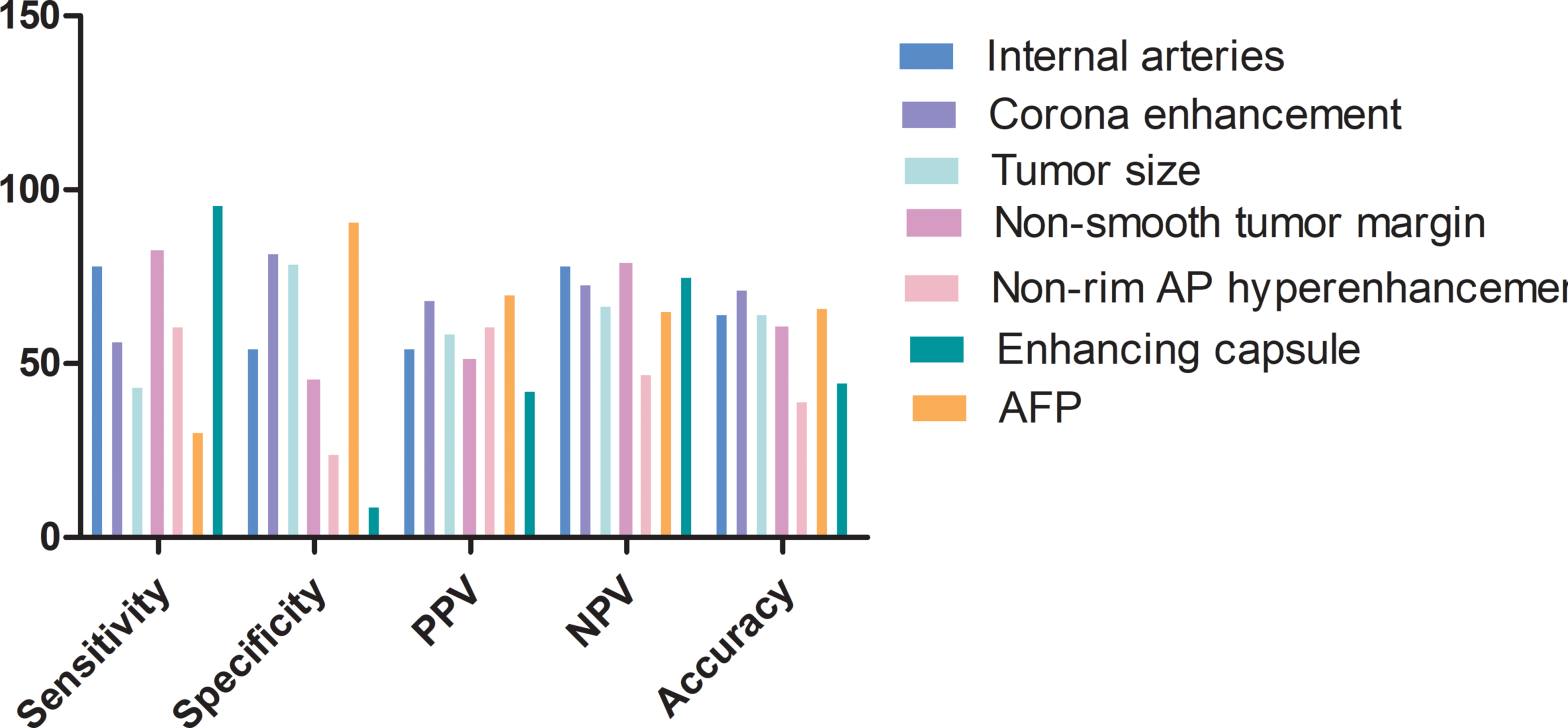
Feature predictive performance for predicting MVI in the test set. AP, arterial phase; PPV, positive predictive value; NPV, negative predictive value.

The predictive power of variables derived from the combined model is shown in **Figure 6**. The highest accuracy variable was corona enhancement. Features with the highest sensitivity was enhancing capsule followed by a non-smooth tumor margin and internal arteries. AFP presented the highest specificity which was followed by corona enhancement.

## 4 Discussion

With the accumulation of knowledge about the prognostic implication of MVI in HCC, it becomes more clinically significant, especially accurate preoperative prediction. Our study selected optimal features including four LI-RADS features (corona enhancement, enhancing capsule non-rim aterial phase hyperehancement, tumor size) and two non-LI-RADS features (internal arteries, non-smooth tumor margin) and AFP. Predictive models of MVI were developed by the SVM approach. Both radiological model and combined model with AFP presented good accuracy and an AUC of MVI prediction in the test set (AUC: 0.775 vs. 0.778, accuracy: 0.714 vs. 0.732, respectively). However, the combined model showed relatively robust accuracy during subgroup analyses in the test set. DCA and the calibration curve also supported the combined model with good performance for MVI prediction.

Based on our prior knowledge and previous literature about MVI and liver tumor (22, 29, 30), we also selected SVM as the classifier and found its good performance of MVI prediction in HCC. Similar to previous studies (10, 11, 31), the serum AFP level is related to the prevalence of MVI. The combined model through adding AFP to the radiological model improved predictive performance, which was reported by previous studies (14, 32, 33). Although we did not find a significant difference between radiological model and combined model, the combined model with AFP recognized MVI false-negative patients predicted by the radiological model in LR-M patients (2 (66.7%)). Therefore, we inferred that it may result from a limited sample size. Notably, Yu et al. also reported that AFP was considered as a suitable biomarker for MVI false-negative patients in conventional pathological protocols (34).

The predictive model that is to be transportable into clinical practice is the final purpose, which is the benchmark indicator assessing its clinical power. Prior studies demonstrated that wide resection (>1 cm), anatomical resection, and intraoperative electron radiotherapy could improve the survival rate of HCC patients along with MVI (35–37). Although LR-M features are not typical features of HCC, HCC with LR-M features have been found to have a significant association with poor prognosis and aggressive behavior (38, 39). To prioritize the allocation of MVI risk probability in LR-M category patients, we defined the 95% specificity threshold in the training set. In the LR-M subgroup, the high specificity threshold could get a specificity of 100% and a PPV of 100%, which may contribute to those high risk for MVI for further treatment. The high-sensitivity threshold could yield an NPV of 100% in the LR-4/5 subgroup. The low-risk patients could avoid biopsy and be considered as the potentially eligible patients for liver transplant.

Regarding LI-RADS features, we selected corona enhancement and major features. Corona enhancement is an LI-RADS ancillary feature for MVI prediction in HCC, which is consistent with previous findings of enhanced MRI (12, 14). Prior studies showed that the reduction in portal flow caused by microscopic tumor thrombin blocking tiny portal vein branches around the tumor caused compensatory hyperperfusion in the AP, which led to corona enhancement (40, 41). Some radiomics studies about MVI prediction focus on the value of the peritumoral area (21, 42). Corona enhancement was the highest accuracy feature which indicates the importance of the peritumoral area. Major LI-RADS features included the enhancing capsule and non-rim enhancement in AP and tumor size in our model. The enhancing capsule and enhancement pattern have remained controversial currently. Some studies suggested that the radiological capsule (22, 23, 43, 44) was a predictor of MVI presence. However, a meta-analysis about enhanced MRI features revealed that the enhancing capsule was not a predictor of MVI (12). Our study was consistent with Xu et al. (22), who obtained a capsule with high sensitivity and low accuracy of MVI prediction, similar to non-rim enhancement in the arterial phase. Wei et al. also reported a typical enhancement pattern which was an independent factor of MVI presence. However, Zhou et al. and Hong et al. showed that rim enhancement in AP or LR-M features was a marker for MVI prediction (12, 32). Thus, those two features need more external data to assess their power of MVI identification. In addition, we also discovered a link between tumor size and MVI. The presence of MVI was also associated with bigger tumor size (>5 cm) according to enhanced MRI (DOR: 5.2 (3.0–9.0) by pooled analysis of inconsistent data (12). Large tumor size was also shown in prior research to be a reliable indicator of MVI based on pathologic results (45, 46), and it was also thought to be a predictor of a poor prognosis (46). A further predictor of vasculature enclosing tumor cluster (VETC) style in HCC was larger tumor size (>5 cm) (47, 48). VETC, a potent pathological style that affects prognosis, was linked to MVI’s frequent existence (48).

We found two non-LIRADS characteristics including internal arteries and a non-smooth margin. Internal arteries were revealed to be the feature with the high sensitivity in our study. Internal arteries predicted on enhanced MRI could be a separate determinant of MVI, according to Jiang et al. (14). Macrotrabecular massive hepatocellular carcinoma, which is regarded as an aggressive form of HCC and commonly has MVI and a poor prognosis, was associated with internal arteries (49–51). Studies also show that internal arteries may serve as a radiogenomic marker for cell growth and matrix penetration (52). The non-smooth margin exhibited a significant relationship with MVI, along with the highest NPV among predictors in our study. The outcome is the same as enhanced MRI (14). Research has shown that tumors with a non-smooth margin were mainly presented in three types of tumors: single nodular type with extranodular growth, confluent multinodular type, and invasive type, which displayed high prevalence of MVI (53). They have suggested that anatomic resection should be employed to reduce the recurrence.

In addition, our study did not find nodule-in-nodule architecture related to MVI, which is different to previous enhanced MRI research (16). The proportion of patients with nodule-in-nodule architecture presented an obvious difference (our study: 1 (0.4%) patients vs. Wei et al.: 49 (44.1%) patients), which may affect the result (16). Similarly, the different ratios of patients with multifocality tumor between our study and Jiang et al.’s study may lead to inconsistent results of MVI prediction (our study: 13 (4.7%) patients vs. 125 (39%) patients) (12).

Our study has some limitations. Firstly, it was a retrospective study whose bias may inevitably exist. Future studies could perform a multicenter prospective study to validate our results. Secondly, our study included mainly HBV-related patients, which may be limitedly applied in patients by other etiologies. More studies are required to explore the application of the model. Thirdly, a gender difference was found between MVI-positive patients and MVI-negative patients in the training set and test set. However, gender did not drive the outcome that no difference exists between correctly and incorrectly predicted MVI patients. Fourth, we did not compare the performance of enhanced CT-based versus enhanced MRI-based LI-RADS features about MVI identification head by head, and further study on the comparison is required in the future. Finally, our research presented no significantly different accuracy and AUC between the radiological model and combined model. More studies with larger samples should be further conducted.

In conclusion, a combination of four LI-RADS features (tree major features, corona enhancement) and two non-LI-RADS features (internal arteries, non-smooth margin) and AFP could be applied as preoperative biomarkers for predicting MVI through a machine learning approach. Furthermore, its good performance in the subgroup by the LI-RADS category may help optimize the management of HCC patients.

## Data availability statement

The raw data supporting the conclusions of this article will be made available by the authors, without undue reservation.

## Ethics statement

The studies involving human participants were reviewed and approved by the ethical committees of the Beijing Youan Hospital, Capital Medical University. Written informed consent for participation was not required for this study in accordance with the national legislation and the institutional requirements.

## Author contributions

XY: conceptualization, data curation, formal analysis, investigation, software. Methodology, visualization, original draft writing. GS: software, review, and editing. JL: review and editing, validation. BL: methodology, validation. CC: validation. DZ: formal analysis, supervision, review and editing, HL: conceptualization, formal analysis, supervision, review and editing. All authors contributed to the article and approved the submitted version.

## Funding

This work was supported by the National Natural Science Foundation of China (grant no. 61936013) and the Beijing Natural Science Foundation (7212051).

## Conflict of interest

The authors declare that the research was conducted in the absence of any commercial or financial relationships that could be construed as a potential conflict of interest.

## Publisher’s note

All claims expressed in this article are solely those of the authors and do not necessarily represent those of their affiliated organizations, or those of the publisher, the editors and the reviewers. Any product that may be evaluated in this article, or claim that may be made by its manufacturer, is not guaranteed or endorsed by the publisher.

## Supplementary material

Supplementary Figure 1 Predictive performance of radiological model and combined model for MVI prediction in HCC patients in the training set and validation set **(A–F)**. Receiver operating characteristic (ROC) curves of radiological model and combined model in the **(A)** training set, **(B)** validation set. Decision curve graphics (DCA) of radiological model and combined model in the training **(C)** and validation set **(D)**. Calibration curve graphics by tertiles of predicted risk based on the radiological model and combined model in training set **(E)** and validation set **(F)**. HCC, hepatocellular carcinoma; MVI, microvascular invasion.
