# Supplementary material for: Predictive machine learning model for microvascular invasion identification in hepatocellular carcinoma based on the LI-RADS system
Source: Front Oncol. 2022 Nov 8;12:1021570. doi: 10.3389/fonc.2022.1021570 (PMC9686848; doi:10.3389/fonc.2022.1021570)
Supplement: Supplementary Figure 1 — Predictive performance of radiological model and combined model for MVI prediction in HCC patients in the training set and validation set (A–F). Receiver operating characteristic (ROC) curves of radiological model and combined model in the (A) training set, (B) validation set. Decision curve graphics (DCA) of radiological model and combined model in the training (C) and validation set (D). Calibration curve graphics by tertiles of predicted risk based on the radiological model and combined model in training set (E) and validation set (F). HCC, hepatocellular carcinoma; MVI, microvascular invasion. [file DataSheet_1.zip › Supplemantary file/Supplementary Table 2.docx]

**Supplemental Table 2** The definition of imaging features in detail.

| **Imaging feature** | **Definition** |
| --- | --- |
| Tumor number | Number of definite intrahepatic HCC with characteristic enhancement pattern(single vs multiple). |
| Tumor size | Measured as the maximum outer-edge-to-outer-edge dimension of the tumor(smaller than 50 mm and larger than 50 mm of the largest tumor) |
| Non-smooth tumor margin | Tumors with irregular  margin and budding part on the edge of tumor in the transverse and/or coronal imaging |
| Internal arteries | The persistence of internal arterial enhancement within the tumor in the arterial phase |
| Peritumoral hypoattenuating halo | A rim of low-density  partially or completely circumscribing the tumor on peritumoral portal venous phase |
| Tumor-liver difference | A focal or peripheral sharp transition of low-density between the tumor and adjacent liver parenchyma in arterial phase |
| Non-rim arterial phase hyperenhancement | Non-rim like enhancement of HCC lesion relative to liver parenchyma in arterial phase |
| Non-peripheral ‘washout’ | Nonperipheral visually evaluated temporal enhancement in whole or in part comparing liver parenchyma in portal phase or delayed phase |
| Enhancing capsule | Smooth, uniform, sharp border around most or all of tumor visible as enhancing rim in portal phase or delayed phase |
| Corona enhancement | The existence of arterial-enhanced region adjacent to the tumor border in the arterial phase, which later presented isodense compared with the background liver parenchyma on CT images in the delayed phase images |
| Nodule-in-nodule architecture | Presence of smaller inner nodule within and having different imaging features than larger outer nodule |
| Mosaic architecture | Presence of randomly distributed internal nodules or  compartments, usually with different imaging features |
| Blood products in mass | Intralesional or perilesional hemorrhage in the absence of biopsy, trauma or intervention |
| Targetoid feature | The presence of at least one feature next: rim arterial enhancement, peripheral “washout”, delayed central enhancement |
| Intratumor necrosis | The presence of unequivocal intralesional necrosis with low-density |
