# Supplementary material for: Predictive machine learning model for microvascular invasion identification in hepatocellular carcinoma based on the LI-RADS system
Source: Front Oncol. 2022 Nov 8;12:1021570. doi: 10.3389/fonc.2022.1021570 (PMC9686848; doi:10.3389/fonc.2022.1021570)
Supplement: Supplementary Figure 1 — Predictive performance of radiological model and combined model for MVI prediction in HCC patients in the training set and validation set (A–F). Receiver operating characteristic (ROC) curves of radiological model and combined model in the (A) training set, (B) validation set. Decision curve graphics (DCA) of radiological model and combined model in the training (C) and validation set (D). Calibration curve graphics by tertiles of predicted risk based on the radiological model and combined model in training set (E) and validation set (F). HCC, hepatocellular carcinoma; MVI, microvascular invasion. [file DataSheet_1.zip › Supplemantary file/Supplementary Table 5.docx]

**Supplementary Table 5 Predictive performance of Radiological model and Combined model for MVI in the training and validation set.**

|  | **Radiological model** | | **Combined model** |
| --- | --- | --- | --- |
| **Training Set** | |  |  |
| AUC | | 0.795 | 0.872 |
| Sensitivity | | 0.63 | 0.73 |
| Specificity | | 0.79 | 0.86 |
| PPV | | 0.69 | 0.78 |
| NPV | | 0.75 | 0.81 |
| Accuracy | | 0.725 | 0.802 |
| **Validation Set** | |  |  |
| AUC | | 0.793 | 0.756 |
| Sensitivity | | 0.52 | 0.65 |
| Specificity | | 0.85 | 0.79 |
| PPV | | 0.71 | 0.68 |
| NPV | | 0.72 | 0.76 |
| Accuracy | | 0.714 | 0.732 |
